# Planning multi-arm screening studies within the context of a drug development program

**DOI:** 10.1002/sim.5787

**Published:** 2013-03-26

**Authors:** James M S Wason, Thomas Jaki, Nigel Stallard

**Affiliations:** a Hub for Trials Methodology Research, MRC Biostatistics UnitCambridge, U.K.; b Medical and Pharmaceutical Statistics Research Unit, Department of Mathematics and Statistics, Lancaster UniversityLancaster LA1 4YF, U.K.; c Warwick Medical School, University of WarwickCoventry, U.K.

**Keywords:** multi-arm multi-stage trials, optimal design, phase II trials, screening trials

## Abstract

Screening trials are small trials used to decide whether an intervention is sufficiently promising to warrant a large confirmatory trial. Previous literature examined the situation where treatments are tested sequentially until one is considered sufficiently promising to take forward to a confirmatory trial. An important consideration for sponsors of clinical trials is how screening trials should be planned to maximize the efficiency of the drug development process. It has been found previously that small screening trials are generally the most efficient. In this paper we consider the design of screening trials in which multiple new treatments are tested simultaneously. We derive analytic formulae for the expected number of patients until a successful treatment is found, and propose methodology to search for the optimal number of treatments, and optimal sample size per treatment. We compare designs in which only the best treatment proceeds to a confirmatory trial and designs in which multiple treatments may proceed to a multi-arm confirmatory trial. We find that inclusion of a large number of treatments in the screening trial is optimal when only one treatment can proceed, and a smaller number of treatments is optimal when more than one can proceed. The designs we investigate are compared on a real-life set of screening designs. Copyright © 2013 John Wiley & Sons, Ltd.

## 1. Introduction

Bringing a drug to market is a long, expensive process 1 requiring multiple clinical trials of various sizes. Generally, clinical trials can be divided into ‘screening’ and ‘confirmatory’ studies. The purpose of screening trials is to gain information about a treatment, such as its side-effects or efficacy, and to decide whether further investigation is warranted. The purpose of a confirmatory trial is to prove to an independent observer that the treatment should be licensed. A question of interest is whether changing the way screening trials are conducted could improve the efficiency of the drug development process as a whole.

The problem of designing screening trials of a single new treatment to maximize the efficiency of a drug development program is discussed by Stallard 2. The problem is considered from the view of a large funder of clinical trials, for whom many treatments are available for testing. Each treatment is tested in a screening trial and, if the screening trial is successful, a confirmatory trial. Treatments are tested sequentially until one succeeds at the confirmatory trial. When the efficiency of the overall drug development process is considered, it has been found that conducting small screening trials on a larger number of treatments is efficient 3,4.

In this article, we extend methods in Stallard 2 to investigate how screening trials that test multiple new treatments should be designed. Whitehead 3 recommends that if there are a limited number of patients and a number of treatments, allocating a small number of patients to a large number of treatments will generally increase the probability of finding a successful treatment. We investigate this idea further and explore optimal design of screening trials to minimize the expected number of patients recruited before a successful treatment is confirmed. In this article, we will also explore how multi-arm screening trials should be designed, including the important question of how many arms should be included in multi-arm screening trials, and multi-arm multi-stage (MAMS) confirmatory trials to maximize the efficiency of the drug development process as a whole.

MAMS trials are ones in which multiple experimental treatments are tested within the same trial with a common control group. Efficiency is gained from the shared control group as well as from interim analyses allowing early dropping of ineffective arms. There are several examples of real MAMS trials, including the Medical Research Council Systemic Therapy in Advancing or Metastatic Prostate cancer: Evaluation of Drug Efficacy trial (STAMPEDE) 6, which uses the methodology described in Royston *et al.* 7, and the TelmisArtan and InsuLin Resistance in HIV (TAILoR) trial, using the methodology described in Magirr *et al.* 8. In addition, there is a large literature on adaptive trials that start with multiple treatments and select treatments to continue with at an interim analysis 9–15. Unlike these designs, we consider separate trials for the selection stage and the confirmatory stage, with focus on designing the selection trial to optimize the drug development process as a whole.

We investigate two types of screening design for comparing multiple treatments: (1) the *top-treatment design*, in which if the test statistic of the most successful treatment at the screening stage is above a threshold, then that treatment passes on to a confirmatory trial in which it is tested against a control treatment; and (2) the *all-interesting-treatments design*, in which all treatments with test statistics above a threshold go on to a multi-arm confirmatory trial. We consider the optimal design of the screening trial in terms of the number of treatments simultaneously tested, the sample size per treatment, and the test statistic threshold for which a confirmatory trial takes place. We also consider how the optimal screening design differs when the confirmatory trial is multi-stage (allowing early stopping for futility or efficacy).

As discussed further in Section 2, the setup used in this article assumes that an inexhaustible number of treatments is available, and that each treatment has an independent treatment effect. A single pharmaceutical company would rarely have several distinct treatments available for testing for the same indication. However, a publicly funded trial such as the STAMPEDE trial 6 may be in a position to compare distinct treatments from several companies. In addition, there are other scenarios where the setup used in this article is applicable. Firstly, the Cocaine Rapid Efficacy Screening Trial (CREST), described in Leiderman *et al*. 16 is a highly relevant example that we look at in Section 4. Secondly, the ‘treatments’ may represent different doses of the same treatment or different treatment combinations. In both cases, the number of treatments available for testing would be much higher than the number of distinct drugs. In the former case, the treatment effects would generally be correlated because of a dose–response relationship; however, in some cases a monotone dose–response relationship may be thought to be implausible, such as in the TAILoR trial (or in cases where higher doses may be less well tolerated no dose–response relationship may exist at all). In these cases, the assumption of independence of treatment effects may be realistic. In the latter case, there may be some correlation between treatment combinations containing the same treatment, but again an assumption of independent treatment effects would likely be realistic. An example where there are a large number of possible treatment combinations available for testing is in tuberculosis 17. A fourth scenario where these assumptions may be realistic is in non-drug trials, where there may be virtually unlimited potential policy interventions awaiting testing. In all these scenarios, the results described in this article would be useful.

## 2. Methods

### 2.1. Multiple arm top-treatment design trials with single-stage confirmatory trials

We first consider screening designs where only the top performing treatment may proceed to a confirmatory trial. The trial design is as follows: at the screening stage, *K* new treatments and a control treatment are each allocated *n*_1_ patients. The treatment response of the *j*th patient on the *i*th treatment (*i* = 0 for the control treatment) is denoted as *X*_*ij*_, and assumed to be distributed as 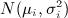. Let 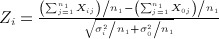, *i* = 1, … ,*K* denote test statistics comparing the active treatments to the control treatment. If max{*Z*_1_,…,*Z*_*K*_} > *c*_1_, where *c*_1_ is a design parameter, then the corresponding top performing treatment is tested against the control treatment in a confirmatory study. The confirmatory trial is assumed to be fixed in size, and its design does not depend on the results of the screening trial. This setup is similar to the design of Thall *et al.* 10, with minor differences including a control group in the screening phase and a normally distributed endpoint instead of a binary endpoint.

Without loss of generality, the mean effect of the control treatment is set to 0, with *μ*_*i*_ representing the mean effect of the *i*th active treatment. The vector of treatment effects (*μ*_1_, … ,*μ*_*K*_) is denoted ***μ***.

The probability of a particular set of *K* new treatments resulting in a successful confirmatory study is


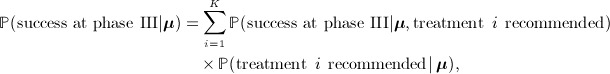


where ‘treatment *i* recommended’ means that treatment *i* passes the screening trial. The event (treatment *i* recommended) is equivalent to (*Z*_*i*_ > *c*_1_ and *Z*_*j*_ < *Z*_*i*_  ∀ *i* ≠ *j*). If all new treatments have the same variance, *σ*^2^, then this is equivalent to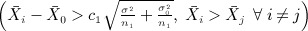, where 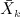 is 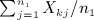.

Because each term depends on 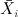, we can integrate the conditional probability of selecting treatment *i* given 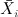 over the distribution of 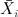 (*N* (*μ*_*i*_,*σ*^2^ / *n*_1_)):


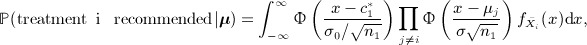


where 
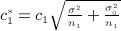
, and 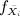 represents the pdf of 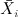 
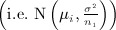
.

We assume that *μ*_1_, … ,*μ*_*K*_ have independent normal prior distributions, with mean *m*_0_ and variance 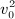, so that the probability of recommending each treatment is identical. The expected probability of recommending treatment *i* can be shown (see supplementary material)‡ to be


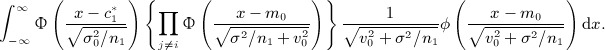
1

We define *P*_1_ as a binary random variable that takes value 1 if and only if a confirmatory trial is conducted, and *P*_2_ as a binary random variable that takes value 1 if and only if a confirmatory trial is conducted and is successful. The expected probability of conducting a confirmatory study, 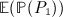, is then *K* times the quantity in Equation (1), which can be found quickly through numerical integration.

Next, we derive the expected probability of a treatment proceeding to the confirmatory trial and subsequently being shown to be significantly more effective than the control (henceforth referred to as ‘phase III success’). This quantity uses the power function of the confirmatory study, which can be written as 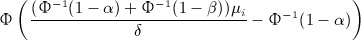 2, where *δ* is the standardized clinically relevant difference used for powering the confirmatory study, *α* is the type-I error rate used, and 1 − *β* is the power. This assumes confirmatory trials are powered independently of the results from the screening trial and so are always of fixed size. This is consistent with the definition of a clinically relevant difference, which should not depend on the previous results but be the treatment effect that would be desirable to detect.

The expected probability that treatment *i* passes the screening trial and is successful at phase III is


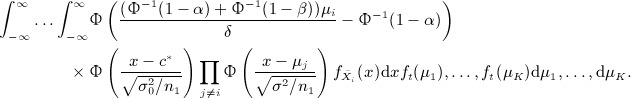
2

As before, terms involving the 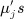 for *i* ≠ *j* can be integrated out, giving the same normal pdf as before. Due to the extra term involving *μ*_*i*_, further simplification is not possible. Equation (2) becomes


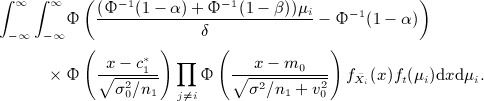
3

For identical prior distributions, the expected probability of a set of *K* treatments leading to a successful phase III trial, 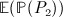 is *K* times the quantity in Equation (3).

Evaluating Equation (3) requires a two-dimensional numerical integration, for which we use the multivariate integration tools provided in the R2Cuba library 18 in R 19.

The expected number of patients recruited up to and including the first phase III success (expected sample size (*ESS*)), as used in Stallard 2, is given by 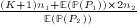, where *n*_2_ is the number of patients recruited per arm in the confirmatory study.

For each value of *K*, we search for the optimal (non-integer) *n*_1_ and *c*_1_ that minimizes *ESS* through use of a search technique such as the Nelder–Mead algorithm 20. We then find the optimal *c*_1_ values when using the floor and ceiling integers of the optimal *n*_1_. The design that gives the lowest *ESS* over all investigated values of *K* is chosen as the optimal screening design.

### 2.2. Multiple arm all-interesting-treatments trials with single-stage confirmatory trials

For the case in which all treatments that pass the threshold progress to the confirmatory trial, the trial design is as follows: at the screening stage, *K* new treatments and a control treatment are each allocated *n*_1_ patients. Test statistics comparing each new treatment to the control treatment, *Z*_1_, … ,*Z*_*K*_, are calculated. Each treatment for which the test statistic is above *c*_1_ advances to a confirmatory study. In the confirmatory study, *n*_2_ patients are assigned to each new treatment and the control treatment. A new treatment is declared effective if the corresponding test statistic is above *c*_2_. The values of *n*_2_ and *c*_2_ depend on the number of new treatments tested in the confirmatory trial. The value of *c*_2_ is chosen so that the family-wise error rate is controlled at *α*. The family-wise error rate is the probability of recommending any ineffective treatment and is a relevant quantity to control in multi-arm trials 21. The value of *n*_2_ is chosen so that the power to detect superiority of a particular new treatment, when its effect is *δ* and the effects of all other new treatments are 0, is equal to 1 − *β*.

Given a specific treatment effect vector ***μ***, the probability of a set of treatments not resulting in a phase II can be conditioned on the number of treatments that pass the screening trial:


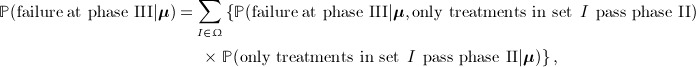
4

where *I* represents a subset of treatments 1, … ,*K* that pass the screening trial, and Ω represents the set of all possible subsets of treatments. Note that because priors of all treatments are assumed to be identical, the probability 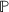
(only treatments in set *I* pass phase II | ***μ***) is identical for all sets *I* with the same size. Note also that there are 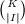 sets with size | *I* | . After integrating over identical prior distributions for each treatment effect, Equation (4) becomes


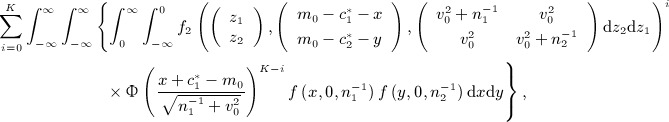
5

where *f*_2_(*z*,*μ*,Σ) is the pdf of a bivariate normal random variable with mean *μ* and covariance matrix Σ (see supplementary material). The inner integral can be efficiently evaluated using the method of Genz and Bretz 22. For the outer integral, which is two-dimensional, we again use the R2Cuba library.

The *ESS* recruited before a phase III success can be written as


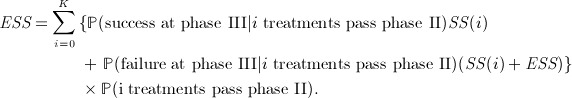
6

The sample size used when *i* treatments pass the screening trial, *SS* (*i*), is equal to (*K* + 1)*n*_1_ + (*i* + 1)*n*_2_(*i*), where *n*_2_(*i*) is the number of patients recruited to each arm in a confirmatory study including *i* new treatments. From the previous text, the equation for *ESS* is


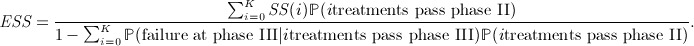
7

### 2.3. Top-treatment screening designs with group-sequential confirmatory trials

By including interim analyses allowing stopping for both futility and efficacy in a confirmatory trial, the average number of patients recruited can be considerably reduced 23. This is at the cost of a slightly increased sample size recruited if the trial does not stop early. Using a group-sequential confirmatory trial may also change the optimal design of the screening trial, as less effective treatments which proceeded to the confirmatory trial can be dropped early.

A group-sequential confirmatory trial changes the formulae used to find the expected sample size until phase III success. This is because the (expected) size of the confirmatory trial depends on the treatment effect and so is no longer independent of the results of the screening trial. We first consider a top-treatment design screening trial with the same notation as in Section 2.1 and a *J*-stage confirmatory trial. Let *ψ* be the random variable representing the treatment selected during the screening trial (*ψ* = 0 represents no treatment is selected) and *χ* ∈ {0,1}be the outcome of the confirmatory trial (*χ* = 1 indicates success). Then the *ESS* until a treatment is confirmed can be found by conditioning on possible values of (*ψ*,*χ*) and noting that sets of treatments tested in subsequent screening studies have independent treatment effects:


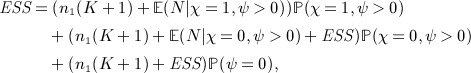
8

where *N* is a random variable representing the *SS* used in the confirmatory trial. This leads to


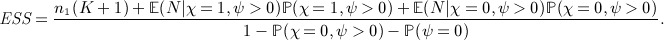
9

The probabilities in the denominator of (9) can be calculated in the same manner as previous sections. The second term in the numerator can be written (because the treatment effect distributions are identical)


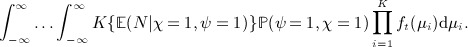
10

The value of 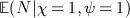 is determined by the probability of the group sequential trial stopping for efficacy at each of the *J* analyses at *μ*_1_. We denote these probabilities as 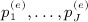. The value of 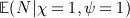 is then equal to 
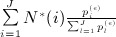
, where *N*^ * ^(*i*) is the *SS* of the group-sequential design, were it is to stop at analysis *i*.

Through use of the same methods in Section 2.1, the number of integrations in Equation (10) can be reduced to give


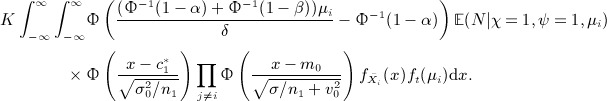
11

The third term in the numerator of Equation (9) can be calculated in a similar way. This leads to a computationally feasible method to calculate the *ESS*.

### 2.4. Multi-arm multi-stage confirmatory trials

If one wishes to allow more than one new treatment to progress to a multi-stage confirmatory trial, as in the designs described in 7 and 8, then it is more difficult to find the *ESS* until phase III success analytically. This is because each treatment in the confirmatory trial has a unique treatment effect, and so the number of integrations required to find the probability of success at phase III (and the *ESS* of the phase III trial) cannot be reduced as before. Thus, any search technique, in which numerical integration is used, to find the optimal *c*_1_ and *n*_1_ is likely to be computationally infeasible.

Instead we use a simulation technique in which a large set of simulated datasets is generated and reused. In the supplementary material, we describe this procedure and apply it to the single-stage confirmatory trial to validate it, which gives the correct optimal design in comparison with the analytic search technique. The simulation method is used to estimate the probability of the phase III trial taking place; the probability of it being successful, conditional on taking place; and the expected sample size of the phase III trial, respectively when it fails and when it is successful.

To estimate the *ESS* for the given values of *c*_1_ and *n*_1_, one can estimate 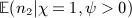, 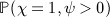, 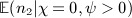, 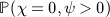, and 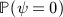, and substitute the values into Equation (9).

## 3. Results

### 3.1. Top-treatment screening trials with single-stage confirmatory trials

Firstly, we look at how the *ESS* until phase III success varies as *K*, the number of new treatments simultaneously tested in the screening stage, increases. Table 1 shows results when the prior mean, *m*_0_, is equal to 0; the prior standard deviation, *v*_0_, is equal to 0.1; 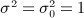; *δ* = 0.25; *α* = 0.025; and 1 − *β* = 0.9. The optimal values for *n*_1_ and *c*_1_ for each value of *K* are shown, together with the *ESS* and the implied family-wise error rate and power of the screening trial. In this context, the family-wise error rate is the probability of a confirmatory trial taking place when all screened treatments have mean treatment effect 0. The power is the probability of a specific treatment progressing to a confirmatory trial when its mean treatment effect is *δ* and all other new treatments have mean treatment effect 0.

**Table I:** Optimal top-treatment design screening trial parameters, expected sample size (*ESS*), the 95 *%* quantile of the sample size (*SS*) (from 5,000,000 simulation replicates), and operating characteristics as *K* varies for *m*_0_ = 0, standard deviation *v*_0_ = 0.1, and clinically relevant difference for confirmatory trial *δ* = 0.25.

*K*	Optimal *n*_1_	Optimal *c*_1_	*ESS*	95 *%* quantile *SS*	Type-I error rate	Power
1	16	0.814	4599	12967	0.208	0.457
2	23	0.676	4236	11830	0.379	0.513
3	25	0.532	4057	11315	0.527	0.525
4	26	0.375	3952	10952	0.657	0.528
5	26	0.214	3886	10722	0.762	0.517
6	25	0.061	3845	10520	0.840	0.493
7	24	− 0.097	3821	10566	0.898	0.466
8	23	− 0.261	3809	10554	0.939	0.439
9	22	− 0.429	3806	10710	0.965	0.412
10	20	− 0.559	3809	10710	0.979	0.376
11	19	− 0.724	3817	10806	0.989	0.350
12	18	− 0.889	3829	10878	0.995	0.326
13	17	− 1.051	3844	10926	0.997	0.303
14	17	− 1.260	3892	11130	0.999	0.291
15	16	− 1.413	3911	11142	1.000	0.271

The *ESS* is minimal at *K* = 9, with the optimal value of *n*_1_ = 22, and the optimal value of *c*_1_ = − 0.429. The latter result is surprising as one would be unlikely to put forward a treatment to a confirmatory trial if it performed worse than the control treatment. In fact, for *K* = 9 and *n*_1_ = 22, one could use *c*_1_ = 0 with just a 0.1 *%* increase in *ESS*. Although the *ESS* is an important quantity to consider, having even an appreciable chance of requiring a huge number of patients is undesirable. Thus, we also examined the 95 *%* quantile of the *SS* required until phase III success, shown in Table 3. This quantity shows a similar pattern to *ESS*, although is minimized at *K* = 6 instead of *K* = 9.

The optimal screening parameters correspond to a family-wise type-I error rate very close to 1 (i.e. if all treatments had the same mean effect as the control treatment, there would be a 0.965 probability of a confirmatory trial taking place). However, note that if all nine treatment effects are from a *N*(0,0.1) distribution, then it is very likely that at least one is better than control.

We next examined the optimal screening parameters as the parameters of the prior distribution vary. Table 2 summarizes the optimal screening trial parameters for different prior treatment effect means, *m*_0_, and prior treatment effect standard deviation, *v*_0_. As *m*_0_ increases, the optimal value of *K* decreases, and the optimal value of *n*_1_ also falls. This is because less precision is required to find a treatment that has a good probability of passing a confirmatory trial as *m*_0_ increases. Also, as expected, *ESS* falls as the mean treatment effect increases.

**Table II:** Optimal top-treatment and all-interesting-treatment designs as *m*_0_ and *v*_0_ vary (for *δ* = 0.25).

*m*_0_	*v*_0_	Optimal top-treatment design							Optimal all-interesting-treatments design											
		*K*	*n*_1_	*c*_1_	*ESS*	95 *%* quantile *SS*	*K*	*n*_1_	*c*_1_	*ESS*	95 *%* quantile *SS*
− 0.1	0.1	12	29	− 0.643	14177	41307	3	36	0.913	18309	53668
− 0.05	0.1	10	26	− 0.468	6907	19456	3	27	1.238	8625	25248
0	0.1	9	22	− 0.429	3806	10710	2	20	0.960	4526	12473
0.05	0.1	8	17	− 0.357	2350	5931	1	12	0.864	2700	7252
0.1	0.1	7	13	− 0.313	1607	3882	1	1	1.904	1754	4965
0	0.05	7	9	− 0.753	12012	35134	1	1	1.860	13276	38966
0	0.075	8	19	− 0.620	6347	17884	1	11	0.729	7304	21347
0	0.1	9	22	− 0.429	3806	10710	2	20	0.960	4526	12473
0	0.125	10	21	− 0.302	2629	7228	2	21	1.037	3146	8331
0	0.15	10	19	− 0.076	2019	5289	2	21	1.109	2417	6156

As the prior standard deviation increases (with *m*_0_ = 0), the optimal values of *K* and *c*_1_ increase. For small values of *v*_0_, there is a lower chance of a treatment with a high treatment effect coming along in a reasonable time, and so the whole procedure is likely to end with a treatment that has a low effect. That is, the phase III trials successes will often be false positives. For larger values of *v*_0_, it is worth putting more resources into the screening trial, as genuinely effective treatments may be found in a reasonable time.

### 3.2. All interesting treatments screening trials with single-stage multi-arm confirmatory studies

We next explore the optimal all-interesting-treatments designs (right-hand side of Table 2). There are interesting differences between the optimal all-interesting-treatments design and the optimal top-treatment designs. The optimal *K* is always lower when multiple treatments can be taken forward. In addition, the optimal value of *c*_1_ is considerably higher than the previous. Both of these factors indicate that fewer phase III trials will be carried out on average when an all-interesting-treatment screening design is used. However, when they are carried out, they have the potential to assess multiple treatments and hence be large.

A second important observation is that *ESS* is higher than for the top-treatment design for every set of prior parameters considered here. This is a surprising result, as one might think that allowing multiple treatments through to a phase III trial would be more flexible. However, these results indicate that the advantages of a greater chance of a particular phase III trial ending in success are outweighed by the larger *SS* required.

For some parameters, for example, when *m*_0_ = 0.1, the optimal screening design is to conduct a screening trial with just one treatment and one patient allocated each to the control treatment and new treatment. The optimal value of *c*_1_ is fairly high (1.904). This would be an extremely unusual screening trial to conduct, and increasing the value of *n*_1_ to a more realistic level does not increase the expected *SS* until phase III success by a great deal.

### 3.3. Top-treatment screening trials with group-sequential confirmatory trials

We used the methods shown in Section 2.3 to investigate the optimal screening designs for different values of *m*_0_ and *v*_0_ when confirmatory trials are designed as two-stage trials by using triangular stopping boundaries 24. Triangular stopping boundaries are used, because they generally have good expected sample sizes for group-sequential 25 and MAMS trials 26.

Table 3 shows the expected *SS* as different numbers of treatments are considered in the screening phase (for *m*_0_ = 0 and *v*_0_ = 0.1). Comparing with Table 3, *ESS* is consistently lower when group-sequential confirmatory trials are used, as expected. The optimal value of *n*_1_ is also always lower, indicating that it is optimal to gather less information on treatments in the screening stage. This seems reasonable as ineffective treatments can always be dropped early in the confirmatory trial. For a similar reason, the optimal value of *c*_1_ is lower when group-sequential confirmatory trials are allowed. These two factors indicate that the screening stage is less important–the first stage of the confirmatory trial serves as an additional screening step. The last difference is that the optimal value of *K* is 8 instead of 9 so for *m*_0_ = 0 and *v*_0_ = 0.1 using slightly fewer treatments is optimal, although the difference in *ESS* between *K* = 8 and *K* = 9 is very small. The 95 *%* quantile of the sample size used is also shown, which, as in the single-stage confirmatory trial case, shows a similar pattern to the *ESS*. The pattern of the 95% quantile is less clear in this case. This is likely because we used fewer simulation replicates to estimate the 95% quantile (compared with the single-stage case), as the group-sequential confirmatory trial increased the amount of computation required for each replicate.

**Table III:** Optimal top-treatment screening trial parameters with two-stage confirmatory trials, expected sample size (*ESS*), the 95% quantile of the sample size (*SS*) (from 500,000 simulation replicates) and operating characteristics as *K* varies for *m*_0_ = 0, standard deviation *v*_0_ = 0.1, and completely randomized design for confirmatory trial *δ* = 0.25.

*K*	Optimal *n*_1_	Optimal *c*_1_	*ESS*	95 *%* quantile *SS*	Type I error	Power
1	8	0.766	3626	10172	0.222	0.395
2	14	0.562	3405	9484	0.427	0.469
3	16	0.385	3290	9128	0.593	0.481
4	16	0.223	3222	8934	0.719	0.464
5	16	0.044	3181	8774	0.820	0.445
6	16	− 0.165	3157	8690	0.898	0.425
7	15	− 0.320	3144	8628	0.939	0.391
8	14	− 0.334	3140	8586	0.950	0.355
9	13	− 0.661	3141	8638	0.982	0.327

Table II in the supplementary material shows the optimal screening designs as *m*_0_ varies and as *v*_0_ varies. A similar pattern is observed as above–the expected *SS* is considerably lower with group sequential confirmatory trials. Additionally, the optimal number of treatments, the optimal value of *n*_1_, and the optimal value of *c*_1_ are all generally lower.

### 3.4. All interesting treatments screening trials with multi-arm multi-stage confirmatory studies

Next we looked at using the all-interesting-treatments screening design with one interim analysis (i.e. a MAMS confirmatory trial). Table II in the supplementary material shows the optimal screening trial designs as *m*_0_ and *v*_0_ vary. As for the top-treatment design, including an interim analysis in the confirmatory trial considerably reduces *ESS*. As before, it generally leads to the same or lower optimal number of treatments in the screening phase, and a lower value of *n*_1_. Unlike the top-treatment design, there is no consistent change in *c*_1_ - sometimes it is increased, and sometimes decreased.

## 4. Case study

As an illustrating example, we consider CREST, an overview of which is given in Leiderman *et al*. 16. CREST was a series of randomized controlled parallel group screening trials. Each trial compared one or more marketed medications to a placebo with the aim of reducing dependence on cocaine in addicts. A total of 19 experimental treatments were screened, although it is possible that with consideration of treatment combinations there would be many more regimens that could be screened. The aim was to recommend treatments to take forward for larger confirmatory trials, testing the same endpoints. Nine normally distributed endpoints were considered across all trials. A type-I error rate of 10% per endpoint, not adjusted for multiple testing, was used, although no formal criteria are listed to determine if a treatment was successful enough to proceed to a larger trial.

We consider the sum of the nine outcomes as the endpoint of interest, which would be used to formally decide which treatments should be tested in confirmatory trials. Although this does not perfectly reflect what was done in reality, we feel it would reflect the spirit of the decision process used. For example, if several endpoints show moderately beneficial effects of an experimental treatment, that treatment would likely be more desirable to test further than a treatment for which a large positive effect had been observed on one endpoint and a large negative effect on another. If each endpoint has some relative importance attached to it, then a different composite endpoint that reflected this, such as a weighted average, could be used.

A series of articles in the same supplement as Leiderman *et al*. reported results of each trial. Included in each article are the means and standard deviations of each outcome for each treatment. We used these results to estimate the average mean treatment effect of an experimental treatment, *m*_0_, the standard deviation of an individual patient's treatment effect, *σ*, and the standard deviation of the treatment effects, *v*_0_. We should note that the latter two quantities depend on the (unreported) correlation between endpoints. We assumed a correlation of 0.2 between each endpoint, which after standardizing to *σ* = 1, gave values of *m*_0_ = − 0.067 and *v*_0_ = 0.165. As in previous sections, we assumed a confirmatory trial would be powered for a 90% probability of detecting a standardized mean difference, *δ*, of 0.25 or more at a one-sided significance level of 0.025.

We found optimal designs for all four classes of screening trial investigated as the number of experimental arms, *K*, varies. Table 4 shows the parameters and expected sample sizes as *K* changes.

**Table IV:** Optimal design parameters for the Cocaine Rapid Efficacy Screening Trial case-study with *m*_0_ = − 0.067, *v*_0_ = 0.165, *δ* = 0.25.

*K*	Single-stage top-treatment				Single-stage all-interesting							Group-seqential top-treatment										Group-sequential all-interesting												
	*n*_1_	*c*_1_	*ESS*	*n*_1_	*c*_1_	*ESS*	*n*_1_	*c*_1_	*ESS*	*n*_1_	*c*_1_	*ESS*
1	20	0.943	3387	20	0.942	3387	13	0.849	2696	13	0.877	2674
2	25	0.885	3038	24	1.039	3186	16	0.783	2449	17	0.926	2540
3	26	0.807	2882	25	1.100	3152	18	0.672	2334	17	0.980	2520
4	26	0.719	2789	25	1.149	3170	18	0.572	2265	17	1.029	2532
5	26	0.622	2727	24	1.181	3196	18	0.461	2219	15	1.124	2553
6	25	0.531	2683	24	1.200	3234	18	0.344	2187	15	1.144	2585
7	24	0.439	2650	22	1.255	3279	17	0.252	2164	15	1.162	2621
8	24	0.329	2627	21	1.306	3372	17	0.117	2148	14	1.215	2661
9	23	0.234	2610	21	1.348	3431	16	0.014	2138	14	1.215	2670
10	22	0.139	2598	21	1.349	3493	16	− 0.122	2131	14	1.228	2742
11	22	0.018	2591	20	1.364	3559	15	− 0.225	2128	13	1.268	2786
12	21	− 0.078	2587	20	1.377	3625	15	− 0.371	2128	13	1.268	2827
13	20	− 0.173	2586	18	1.439	3689	14	− 0.472	2129	12	1.328	2868
14	20	− 0.302	2587	18	1.462	3752	14	− 0.622	2143	12	1.328	2910
15	19	− 0.396	2590	18	1.462	3814	13	− 0.713	2148	12	1.345	2952

For the two top-treatment designs, the optimal number of experimental arms is high, equal to 13 and 11 for single-stage and two-stage confirmatory trials, respectively. The optimal number of arms for the all-interesting-treatments design is 3 in both cases. This broadly agrees with the result in the previous section. In the actual CREST screening studies, a sample size of around 15 per arm was used. The number of experimental arms in each screening study varied between 1 and 4. This parameters are close to optimal if multiple experimental treatments are to be tested in the same confirmatory trial, but much smaller than optimal if just the top treatment goes ahead to a confirmatory trial.

We also investigated the treatment effect of the final recommended treatment for the four optimal screening designs in Table 4. In the case of a multi-arm confirmatory trial that recommends more than one treatment, we recorded the one with the higher final test statistic as the final recommended treatment. We simulated 100,000 replicates for each design. Interestingly, all four designs gave a very similar distribution, with the median treatment effect of the confirmed treatment being around 0.2 in each case. The estimated probability of the final recommended treatment actually being worse than control ranges between 0.01 and 0.05. This is as compared with a per-study one-sided significance level of 0.025 used in the confirmatory study.

## 5. Discussion

In this article we have explored optimal design of multi-arm screening trials. A normally distributed endpoint is assumed to allow analytic formulae to be derived. The methods could be extended to other types of endpoints for which normally distributed test statistics are available, such as binary and time-to-event. The problem can be formulated as a Bayesian decision–theoretic design with the aim to minimize the prior expected loss; the loss function in this case is taken to be the number of patients recruited before a treatment is found successful at the confirmatory trial. We investigated the top-treatment design, similar to that of Thall *et al.* 10, in which a maximum of one treatment may proceed to a confirmatory trial; the second is the all-interesting-treatments design, which allows multiple treatments to proceed to a confirmatory trial. The top-treatment design is more efficient than the all-interesting-treatments design for all scenarios considered, with the optimal design generally including a large number of treatments with a relatively low number of patients per arm and a less strict test statistic threshold determining whether a confirmatory trial should be conducted. Interestingly for some designs the optimal critical value is negative, indicating that even if the best experimental treatment performs worse than the control treatment, it should still be tested in a confirmatory trial. This is clearly not ideal in real life, and shows that considerations other than efficiency should be taken into account in the design of screening trials.

In both classes of design, a control group is included in the screening stage. Theoretically it may be more efficient not to, but the procedure would then be prone to biases that are hard to quantify, such as patient drift. The patients included in the screening stage are also assumed not to be included in the confirmatory trial (as opposed to a seamless phase II/III design). Despite a loss in efficiency, we consider separate screening and confirmatory trials here, as it is a more common approach. It also provides a chance to make changes to the confirmatory trial design and selection of treatment in response to new external information (for example, results from related trials being published). Because of the small number of patients in the screening trials examined here, the loss in efficiency is small.

In Stallard 2, minimizing the expected number of patients until a treatment is successful at a confirmatory trial is equivalent to maximizing the expected number of confirmed treatments found from a finite set of patients. When multiple treatments are tested simultaneously this equivalence is not true. Confirming more than one treatment might be desirable. For example, the different treatments may be more effective in different subgroups or have different side-effect profiles. In the supplementary material, we investigate optimal screening designs when the optimality criterion is the expected number of confirmed treatments per recruited patient. By using that criterion, the optimal all-interesting-treatments design includes more treatments in the screening stage and is closer in efficiency to the top-treatment design. Many other metrics could be of interest, such as different quantiles of the sample size until confirmation trial success distribution, or the probability of a genuinely effective treatment being recommended. We chose to focus on the expected sample size until phase III success as it has a simple interpretation, is comparable between designs, and also reflects the amount of resources needed in the drug development process.

The top-treatment and all-interesting-treatment designs can be expressed as extreme cases of a more general screening design. An alternative, which would be an interesting area for research, would be to include additional treatments in the confirmatory trial if the increase in predictive probability of confirmatory trial success (given the screening data) compensates for the increase in *SS* required. In other words, one would predict the *ESS* of the remaining process given the screening data for different numbers of treatments and choose the most efficient number of treatments to undergo a confirmatory trial.

A number of assumptions are made in this article, as discussed in the parallel-group setting 2. One is that the treatment effects are identically distributed from a normal distribution. This assumption allows analytic formulae to be derived for the expected sample size. If one wanted to find optimal screening designs that allow for different prior distributions, then this could be accomplished using the simulation method. A second is that the same endpoint is used in both the screening and confirmatory trials, which is often not true but was for the CREST trial 16. In the case of different endpoints, the treatment effect on the phase II endpoint would be correlated with the treatment effect on the phase III endpoint. Exploring how optimal screening designs differ when the endpoints used in screening and confirmatory is the subject of future research.

## Footnotes

‡ Supporting information may be found in the online version of this article.
