# Supplementary material for: Planning multi-arm screening studies within the context of a drug development program
Source: Stat Med. 2013 Mar 26;32(20):3424–35. doi: 10.1002/sim.5787 (PMC3882502; doi:10.1002/sim.5787)
Supplement: Supplementary file 1 [file sim0032-3424-sd1.pdf]

Supplementary material for 'Planning multi-arm screening studies within the context of a drug development programme'

James M. S. Wason, Thomas Jaki and Nigel Stallard

1 Derivations of analytical formulae for probability of confirming treatments

1.1 Top-treatment design

Let us assume $\mu_1, \ldots, \mu_K$ have independent prior distributions, all with density, $f_t(.)$. To find the expected probability of recommending treatment i, we integrate over all these distributions:

$$\int_{-\infty}^{\infty} \cdots \int_{-\infty}^{\infty} \Phi\left(\frac{x - c_1^*}{\sqrt{\sigma_0^2/n_1}}\right) \prod_{j \neq i} \Phi\left(\frac{x - \mu_j}{\sqrt{\sigma^2/n_1}}\right) f_{\bar{X}_i}(x) dx f_t(\mu_1) \ldots f_t(\mu_K) d\mu_1 \ldots d\mu_K. \quad (1)$$

For a normal prior distribution with mean m_0 and variance v_0^2, μ_j can be integrated out $\forall j \neq i$:

$$\int_{-\infty}^{\infty} \Phi\left(\frac{x - \mu_j}{\sqrt{\sigma^2/n_1}}\right) f_t(\mu_j) d\mu_j = \int_{-\infty}^{0} \int_{-\infty}^{\infty} f^*(z - \mu_j) f_t(\mu_j) d\mu_j dz, \quad (2)$$

9 where f^* is the pdf for a normal distribution with mean $-x$ and variance $\frac{\sigma^2}{n_1}$. This is the integral of a convolution of two independent normal distributions. The convolution is itself normal with mean $-x + m_0$ and variance $\frac{\sigma^2}{n_1} + v_0^2$, evaluated at z. Therefore equation (2) becomes $\Phi\left(\frac{x - m_0}{\sqrt{\sigma^2/n_1 + v_0^2}}\right)$.

A similar argument can be used to integrate out μ_i:

$$\int_{-\infty}^{\infty} f_{\bar{X}_i}(x) f_t(\mu_i) d\mu_i = \int_{-\infty}^{\infty} f_{\bar{X}_i^*}(x - \mu_i) f_t(\mu_i) d\mu_i, \quad (3)$$

where $f_{\bar{X}_i^*}$ is the pdf of a normal random variable with zero-mean and variance $1/n_1$. Equation (3) is the pdf of the normal distribution with mean m_0 and variance $v_0^2 + \sigma^2/n_1$ evaluated at x. Equation (1) then becomes:

$$\int_{-\infty}^{\infty} \Phi\left(\frac{x - c_1^*}{\sqrt{\sigma_0^2/n_1}}\right) \left\{\prod_{j \neq i} \Phi\left(\frac{x - m_0}{\sqrt{\sigma^2/n_1 + v_0^2}}\right)\right\} \frac{1}{\sqrt{v_0^2 + \sigma^2/n_1}} \phi\left(\frac{x - m_0}{\sqrt{v_0^2 + \sigma^2/n_1}}\right) dx. \quad (4)$$

1.2 All-interesting-treatments design

Given a specific treatment effect vector μ, the probability of a set of treatments not resulting in a phase III success can be conditioned on the number of treatments that pass the screening trial:

$$\begin{aligned}\mathbb{P}(\text{Failure at Phase III}|\mu) = \sum_{I \in \Omega} \{&\mathbb{P}(\text{Failure at phase III}|\mu, \text{only treatnents in set } I \text{ pass phase II}) \\ &\times \mathbb{P}(\text{only treatments in set } I \text{ pass phase II}|\mu)\},\end{aligned}$$

where I represents a subset of treatments $1, \ldots, K$ that pass the screening trial, and Ω represents the set of all possible subsets of treatments. For identical priors of all treatments, the probability $\mathbb{P}(\text{only treatments in set } I \text{ pass phase II}|\mu)$ is identical for all sets I with the same size. There are $\binom{K}{|I|}$ sets with size $|I|$, thus after integrating over identical prior distributions for each treatment effect, the expression becomes:

$$\begin{aligned}&\int_{-\infty}^{\infty} \cdots \int_{-\infty}^{\infty} \mathbb{P}(\text{Failure at Phase III}|\mu) \prod_{j=1}^{K} f_t(\mu_j) d\mu_j \\ =&\int_{-\infty}^{\infty} \cdots \int_{-\infty}^{\infty} \sum_{i=0}^{K} \binom{K}{i} \{\mathbb{P}(\text{Failure at phase III}|\mu, \text{Treatments } 1, \ldots, i \text{ pass phase II}) \\ &\mathbb{P}(\text{Treatments } 1, \ldots, i \text{ pass phase II}|\mu) \prod_{j=1}^{K} f_t(\mu_j) d\mu_j\Bigg\}. \quad (5)\end{aligned}$$

We define $\bar{X}_j^{(1)}$ and $\bar{X}_j^{(2)}$ as the mean response of treatment j in the screening and confirmatory trial respectively.

The event (Failure at phase III | Treatments $1, \ldots, i$ pass phase II) is equivalent to $(\bar{X}_1^{(2)} \leq c_2^* + \bar{X}_0^{(2)}, \ldots, \bar{X}_i^{(2)} \leq c_2^* + \bar{X}_0^{(2)})$, where $c_2^* = c_2\sqrt{\frac{\sigma_0^2}{n_2} + \frac{\sigma^2}{n_2}}$.

Similarly, the probability of event (Treatments $1, \ldots, i$ pass phase II) is equal to

$$\left(\prod_{j=1}^{i} \mathbb{P}(\bar{X}_j^{(1)} > c_1^* + X_0^{(1)}) \prod_{j=i+1}^{K} \mathbb{P}(\bar{X}_j^{(1)} \le c_1^* + X_0^{(1)})\right),$$

where $c_1^* = c_1\sqrt{\frac{\sigma_0^2}{n_1} + \frac{\sigma^2}{n_1}}$.

As previously, we can condition on the means of the control treatment in the screening trial, and in the confirmatory trial. The number of integrations can be reduced with normal priors. For example if $j \le i$, then conditional on $(X_0^{(1)}, X_0^{(2)}) = (x, y)$:

$$\int_{-\infty}^{\infty} \mathbb{P}(\bar{X}_j^{(2)} \le c_2^* + y)\mathbb{P}(\bar{X}_j^{(1)} > c_1^* + x) f_t(\mu_j) d\mu_j$$

can be written as the distribution of a bivariate normal distribution [1]. For $j > i$, then:

$$\int_{-\infty}^{\infty} \mathbb{P}(\bar{X}_j^{(1)} \le c_1^* + \bar{X}_0^{(1)}) f_t(\mu_j) d\mu_j$$

can be written as the tail probability of a univariate normal distribution, as for equation (2). With these simplifications, the ith summand in equation (5) can be written as:

$$\int_{\infty}^{\infty}\int_{\infty}^{\infty}\left\{\int_{0}^{\infty}\int_{-\infty}^{0} f_2\left(\left(\begin{array}{c} z_1 \\ z_2 \end{array}\right), \left(\begin{array}{c} m_0 - c_1^* - x \\ m_0 - c_2^* - y \end{array}\right), \left(\begin{array}{cc} v_0^2 + n_1^{-1} & v_0^2 \\ v_0^2 & v_0^2 + n_2^{-1} \end{array}\right) dz_2 dz_1\right)^i \right.$$
$$\left. \times \Phi\left(\frac{x + c_1^* - m_0}{\sqrt{n_1^{-1} + v_0^2}}\right)^{K-i} f(x, 0, n_1^{-1}) f(y, 0, n_2^{-1}) dx dy\right\}. \quad (6)$$

2 Simulation approach for all-interesting treatment screening designs with multi-arm multi-stage confirmatory trials

2.1 Methods

The simulation procedure, referenced in section 2.4 of the main paper, is as follows:

1. Set $K_{\max}$, the maximum number of treatments that will be considered; m_0, the mean of the treatment effect distribution; v_0, the standard deviation of the treatment effect distribution; and R, the number of replicates.

2. Find the set of MAMS trial parameters for each possible value of K between 1 and K_{max}. We find the stopping boundaries using the triangular test[2], such that the family-wise type-I error rate is α. The sample size per arm per stage is found so that the power to confirm a specific treatment when it is effective and all other new treatment ineffective is 1-β.

3. Generate $R \times K_{\text{max}}$ standard normally distributed variables representing the test statistics of each treatment at the screening trial. Store values in an $R \times K_{\text{max}}$ matrix, S. Transform the matrix so that the pairwise correlation between each column is 0.5 (representing the correlation between z-test statistics using the same controls when the allocation ratio is 1:1).

4. Generate $R \times J \times K_{\text{max}}$ standard normally distributed random variables representing the increment of information for each new treatment at each stage of a MAMS trial. Store values in a $N \times J \times K_{\text{max}}$ three-dimensional array, C. As before, transform the data so that the correlation between variables representing different treatments within each stage is 0.5.

5. Generate $N \times K_{\text{max}}$ N(m_0,v_0^2) random variables, representing the mean treatment effect of each new treatment. Store values in an $R \times K_{\text{max}}$ matrix, μ.

6. For a given value of K, n_1, c_1, convert the screening trial simulated data so that they have the correct mean. That is, Z_{ik}^S, the z-test for the kth new treatment in the ith, is equal to $S_{ik} + \mu_{ik} \times \sqrt{n_1/2}$. For that replicate, count the number of new treatments with test statistic greater than c_1. For those treatments, transform the relevant MAMS trial data so that it has the correct mean and covariance, as described further in Wason and Jaki[3]. For each replicate in which a confirmatory trial takes place, determine whether the confirmatory trial ends in success and the sample size used.

7. For each replicate, one can determine whether a confirmatory trial took place, and if so, its sample size and whether it was successful. Averaging these quantites over all replicates gives the required quantities needed to substitute in to equation (12) in the main paper. The resulting expected sample size is the expected sample size for that value of n_1 and c_1.

8. For each value of K between 1 and K_{max}, use a search technique, such as Nelder-Mead, to search over values for n_1 and c_1 that give the optimal expected sample size.

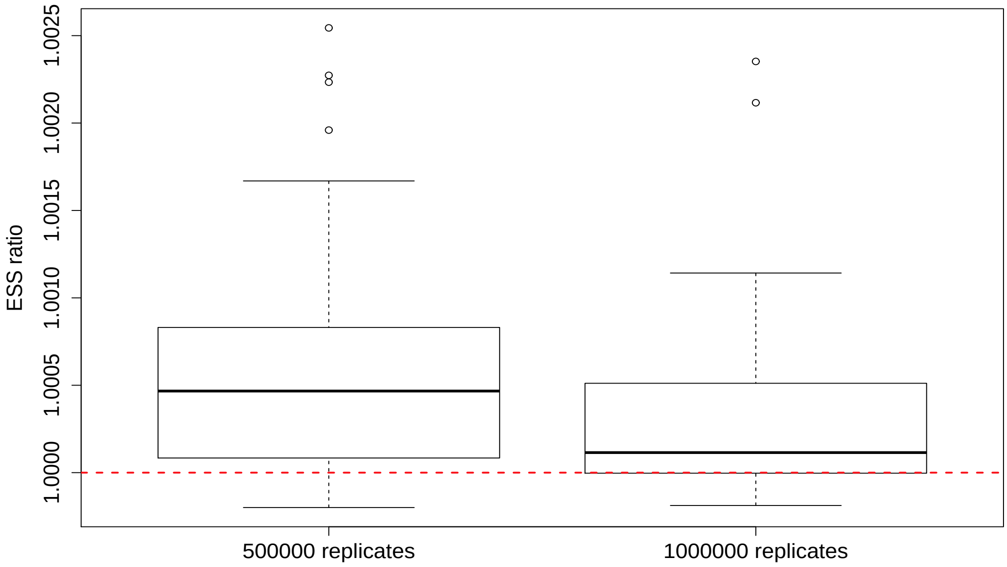

Figure 1: Boxplots showing ratio of 'optimal' expected sample sample size found from simulation method to optimal expected sample size found from using the analytic formulae. Results are for $m_0 = 0$ and $v_0 = 0.1$.

9. For the ceiling and floor integers of n_1, find the optimal value of c_1, and determine the optimal integer n_1 together with c_1.

2.2 Validation

In this section, we examine whether the simulation method gives sensible answers. We implemented the simulation method for the all-interesting-treatments screening trial with one-stage confirmatory trial scenario for which we have analytic formulae to evaluate the expected sample size. We repeated the simulation search process 50 times, each time using the analytic formula to get the true ESS of the found design. We investigated using 500,000 and 1,000,000 replicates in the search process. Figure 1 shows the distribution of the ratios of the expected sample size from each search process compared to the actual 'optimal' ESS from the analytic search process.

The results show that as the number of replicates are increased, the reliability of the process improves. However, even the worse performing case gave an expected sample size within 0.25% of the true optimum. Interestingly, some cases actually gave a better ESS than the one found using the analytic search procedure. This is because numerical integration itself has uncertainty associated with it, and so although analytic formulae

exist, methods for evaluating them are not necessarily fully reliable.

From these results, we concluded that if 10 independent simulation search processes of 1,000,000 replicates each are used, the resulting design should be very-near to the optimal one.

3 Optimal designs when multiple new treatments are of interest

3.1 Rationale

The optimality criterion used in the main paper assumes that finding just one new treatment that passes phase III. In practice, finding more than one new treatment that outperforms control may be of interest, and may lead to more than one new drug being licensed. For example, if the new drugs have different side-effect profiles, or are more effective in different subgroups.

Assuming that finding more than one new treatment is of interest, a different optimality criterion is required. One could maximise the number of treatments confirmed, but this maximum would occur when all treatments proceed to a confirmatory phase. Instead, taking into account the number of patients recruited results in a more realistic optimality criterion. We thus chose to optimise the expected number of treatments confirmed per 1000 patients recruited.

We adapted the simulation technique described above to search for the optimal top-treatment and all-interesting-treatments designs when the confirmatory trial was not group-sequential.

3.2 Results

The optimal top-treatment and all-interesting-treatment designs for $m_0 = 0, v_0 = 0.1$ are shown in table 1.

Design	K	n_1	c_1	Expected number of confirmed treatments per 1000 patients
Top-treatment design	8	23	-0.266	0.265
All-interesting-treatment design	5	26	1.03	0.235

Table 1: Optimal design and expected number of treatments confirmed per 1000 patients recruited for top-treatment and all-interesting-treatment designs. K: optimal number of treatments at pilot stage; n_1: optimal number of patients recruited per arm in screening studies; c_1: test-statistic threshold at which confirmatory trial takes place.

The optimal number of treatments to include for the all-interesting-treatments design is considerably higher in comparison to when minimising the expected sample size until phase III success is of interest. The optimal number is 5 instead of 2, which reflects that confirmatory trials finishing with more than one confirmed treatment will contribute towards increasing the objective function used here, but not to the expected sample size until phase III success. There is less difference in the optimal top-treatment designs (8 treatments instead of 9).

4 Supplementary results

		Optimal top-treatment design				Optimal all-interesting-treatments design			
m_0	v_0	K	n_1	c_1	ESS	K	n_1	c_1	ESS
-0.1	0.1	10	18	-0.725	11252	2	17	0.874	13962
-0.05	0.1	9	16	-0.612	5582	2	13	1.022	6653
0	0.1	8	14	-0.334	3140	2	12	1.074	3621
0.05	0.1	7	11	-0.545	1966	1	6	1.315	2245
0.1	0.1	7	9	-0.430	1344	1	1	1.741	1500
0	0.05	7	4	-0.821	9178	1	1	1.741	9396
0	0.075	7	11	-0.623	5096	1	2	1.522	5786
0	0.1	8	14	-0.334	3140	2	12	1.074	3621
0	0.125	9	15	-0.412	2183	2	12	1.124	2538
0	0.15	10	15	-0.337	1663	2	13	1.195	1949

Table 2: Optimal top-treatment and all-interesting-treatment designs, when two-stage confirmatory trials are carried out, as m_0 and v_0 vary (for $\delta = 0.25$)

References

[1] N. Stallard. Optimal sample sizes for phase II clinical trials and pilot studies. *Statistics in Medicine*, 31:1031–1042, 2012.

[2] J. Whitehead and I. Stratton. Group sequential clinical trials with triangular continuation regions. *Biometrics*, 39:227–236, 1983.

[3] J.M.S. Wason and T. Jaki. Optimal design of multi-arm multi-stage trials. *Statistics in Medicine*, E-published, 2012.
